# Typology, technical efficiency and scale economy of *dibiteries *in Dakar, Senegal

**DOI:** 10.12688/aasopenres.12953.2

**Published:** 2019-12-12

**Authors:** Malik Orou Seko, Walter Ossebi, Gnamien Sylvain Traoré, Andrée Prisca Ndjoug Ndour, Jasmina Saric, Gilbert Fokou, Daouda Dao, Bassirou Bonfoh

**Affiliations:** 1 Ecole Inter-Etats des Sciences et Médecine Vétérinaires (EISMV), Dakar, Senegal; 2 Centre Suisse de Recherches Scientifiques en Côte d’Ivoire (CSRS), Abidjan, Cote d'Ivoire; 3 Université Peleforo Gon Coulibaly (UPGC), Korhogo, Cote d'Ivoire; 4 Swiss Tropical and Public Health Institute, Basel, Switzerland; 5 University of Basel, Basel, Switzerland; 6 Human Science Research Council (HSRC), Cape Town, South Africa; 7 Université Félix Houphouët-Boigny (UFHB), Abidjan, Cote d'Ivoire

**Keywords:** Dibiterie, Data envelopment analysis, Efficiency, Scale economy, Quality, Senegal

## Abstract

Background: In recent years, a profound transformation has been observed in the eating habits of the populations of African cities, induced by accelerated socioeconomic and demographic growth. In Senegal, these changes have manifested in the proliferation of collective informal catering enterprises, such as the ‘ *dibiteries*’, where the roasted meat of sheep is prepared and sold. The rise of the average household income has contributed substantially to increasing levels of meat consumption, leading to the expansion of the *dibiteries*. The purpose of the current work was to evaluate the managerial performance of these establishments in Dakar, Senegal.

Methods: To achieve this, a cross-sectional study was conducted among 152 *dibiteries* using a questionnaire. Efficiency scores were determined via the data envelopment analysis method. The pure technical scores thereby obtained were subsequently used as dependent variables in a Tobit model to identify the socioeconomic determinants of *dibiterie* efficiency.

Results: The resulting average score of the *dibiteries* suggests that the majority are operating inefficiently (79.6%). Moreover, it was demonstrated that this inefficiency seems to be related to scale rather than technical issues. However, few of the *dibiteries* assessed (20.4%) were nevertheless in a situation of constant scale economy. Among the socioeconomic variables tested, experience, leadership (family or individual-run), the ownership status of the restaurant building (own or lease) and the type of workforce (family, recruited, mixed or without) had a significant impact on the efficiency of the establishments.

Conclusions: The scale economy and waste reduction in food production can result in economic gains that can in turn be used in the safety of finished products. Indeed, by following best practices, *dibiteries* can make gains which could be used to invest in good hygiene practices on handwashing, cleaning and disinfecting grilling tools, optimizing work space and training staff.

## Introduction

In Senegal, livestock management occupies nearly two-thirds of the country's agricultural households and constitutes, together with agriculture, the main activity of the rural populations and the main supplier of food and income. The livestock subsector has experienced a real dynamism in recent years, with sustained performances, particularly in the production of meat and milk ( Sénégal, 2018). According to the Ministry of Livestock and Animal Products, almost all meat supply in Senegal in 2015 derives from poultry (36%) and ruminants (i.e. 35% cattle, 14% sheep and 9% goat); representing the protein sources in 21% of all evening meals in urban areas, only surpassed by fish, which accounts for 75% of all animal protein on the dinner plate ( Ndoye *et al*., 2001; Sénégal, 2017). Mutton in particular is the preferred choice in collective catering and religious receptions such as the ‘ *Magal of Touba*’ because of its nutritional value and its socioeconomic and cultural importance. This species is bred for the self-consumption as well as to supply for Muslim festivities.

Introduction of the continuous work day in Senegal in 1992, the devaluation of the CFA franc in 1994, rapid urbanization and the non-appreciation of the common household meals contributed to increasing out-of-home consumption, and significantly modified the dietary habits of the Senegalese population ( Ndoye *et al*., 2001). In contrast to discontinuous work (7 am to 12 noon and 2 to 6 pm), the continuous work schedule (7 am to 5.30 pm) has reduced the length of time reserved for lunch down to 30 minutes. The growing urbanization has further challenged the feasibility of home-based lunches by increasing the distances between home and work base and by placing a strain on public transport systems. Most workers today are consequently forced to eat out-of-home, including those with limited financial means, representing the majority of the urban population. This shift, together with an ever-growing population in Dakar and the improvement of the standard of living, are at the origin of the rise in demand for meat products ( Thornton *et al*., 2007), dairy bars, canteens, fast food suppliers and collective catering enterprises such as the ‘ *dibiteries*’ ( Duteurtre, 2009).

The *dibiteries* specialize in charcoal firewood-roasted mutton and occasionally chicken ( Duho, 2012). The employees working in these informal enterprises are often family members, and they are allocated according to the different tasks necessary to run the business, namely cutting, grill, service and management ( Aw, 1996; Marchand, 2005). The sheep carcasses used in the *dibiteries* in the Dakar region are usually moved from the slaughterhouses by public transport, without being maintained in a cold chain. After cooking, the meat is seasoned with condiments (e.g. pepper, salt, onions) and then wrapped in paper recycled from the packaging of wheat flour or cement ( Aw, 1996; Dione, 2000).

*Dibiteries* are accommodating all the needs of the new urban working population by offering fast and cheap food ‘around the corner’ and have, in addition, a strong sociocultural attraction, owing to the significance of sheep in Senegalese societies. They also represent a public health nuisance to health authorities, and a major personal health risk to the consumer population, by serving products at sub-standard hygiene conditions. It has been previously shown that consumers had a one-in-two chance (50.5%) of acquiring a microbial meat contaminant during the consumption of braised meat in the *dibiteries* of the Dakar region ( Yougbare, 2014). In addition to putting the consumer’s health at risk, the underlying hygiene deficit has a negative impact on the quality of the meat, causing a loss of market and income for their promoters.

The last study conducted on the profitability of *dibiterie* establishments in the Dakar region, was conducted in 2005 ( Marchand, 2005) pointing out that profits are still being generated by the braised meat trade. However, it further demonstrated that these profits are shared within the family, instead of being used to expand their business by improving the framework and product quality and reduce the health risks to the consumer.

To find out if the economic situation has changed since, the present work has been conducted with the aim to characterize and to assess the technical efficiency and the scale economy of *dibiteries* in Dakar, and to identify strategies to improve the quality of braised meat through good efficiency management.

## Methods

### Study area

**Figure 1.**
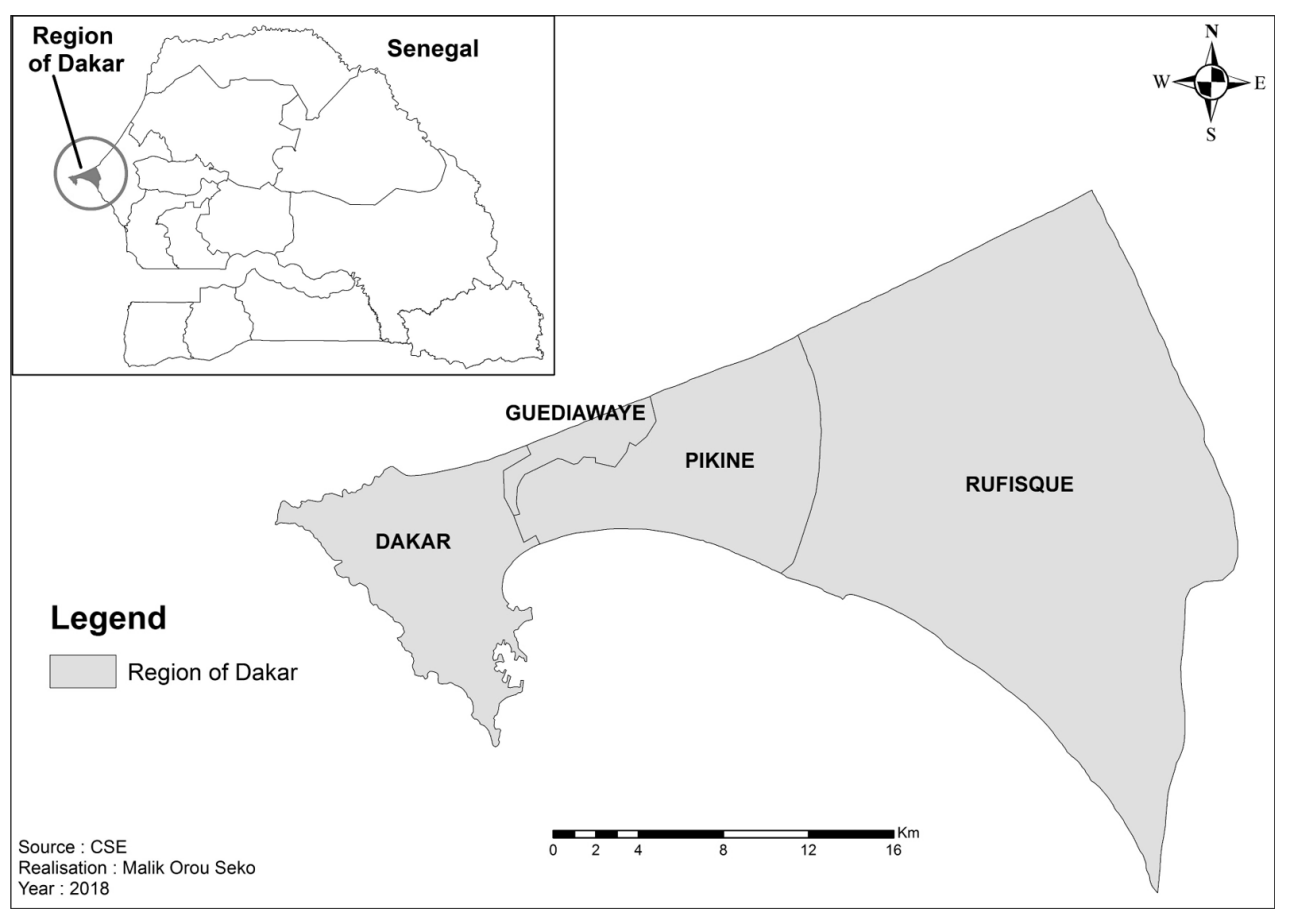
Map of Dakar region.

The study was conducted in four Departments of Dakar region, Senegal, namely Dakar, Guédiawaye, Pikine and Rufisque ( Figure 1). The capital Dakar was chosen because it represents the main center of demand for food products, concentrating one-quarter of the national population. In addition, the purchasing power of consumers is high, compared with other regions ( Mankor, 2009; Sénégal, 2014) and livestock from across the country converge in the Dakar region. In 2013, the National Agency of Statistics and Demography counted a total of 936,239 ruminants (125,009 cattle, 594,892 sheep and 216,338 goats) entering the Dakar area. Almost all animals going through this pathway are destined for butchery. The total number of slaughters recorded in 2013 at the Slaughterhouse Management Company of Senegal (SOGAS) and in the Department of Rufisque amounted to 27,552.259 tons of meat. In Rufisque, the slaughter of cattle is more frequent than small ruminants (60,347 vs 7175). However, at SOGAS level which covers the departments of Dakar, Pikine and Guédiawaye, 513,706 small ruminants were slaughtered (5,662,116 kg) vs 67,810 cattle (10,169,354 kg) ( Senegal, 2014).

### Sampling and data collection

The *dibiteries* were chosen using an empirical accidental sampling approach. This is a non-probabilistic method in which individuals are retained when encountered until the desired number of individuals is obtained. This method was chosen not only because of the absence of a list of *dibiterie*s at the level of the departmental livestock services, but also taking into account the unwillingness of *dibiteries* owners to open easily to the investigators. It was decided that 200 *dibiteries* would provide a representative sample, 50 establishments in each Department.

The establishments chosen were those that are routinely inspected by the Livestock Service Officer, and that agreed to participate in the survey. Thus, the recruitments of the *dibiteries* were carried out with the support of the veterinary inspection officers. However, the establishments of *dibiteries* that did not routinely inspected by the officers of veterinary inspection services have not been investigated. The interviews were conducted from January to April 2015 in semi-direct mode in French or Wolof, using a questionnaire (an English translation is provided as extended data ( Orou-Seko *et al*., 2019b)). Quantitative variables included the products purchased, sales and income, labour, equipment and transportation. The qualitative variables covered socio-demographic data on the *dibiterie* owner and his enterprise. As part of the latter, the origin of the *dibiterie* tenant and the technique of the production of the braised meat were assessed in agreement with the actors of this sector to classify the *dibiteries*.

### Ethical approval

The study was carried out with the permission of the Ministry of Livestock of Senegal (Authorization N° 1611) and the oral informed consent of the participants for using information that they have given, also for their publication. Prior to data collection, participants were informed of the merits of the study in order to obtain their oral informed consent. Finally, the survey participants were informed of confidentiality and anonymity, and that the results will only be used for research and strategic decisions.

### Variables

***Input variables.*** The production of braised mutton in *dibiteries* is based on eight factors, including (i) labour, (ii) combustible (wood and coal), (iii) water and electricity, (iv) condiments, (v) sheep, (vi) transportation (service), (vii) amortization and (viii) ‘other’ charges. The number of people hired in the *dibiteries* was recorded to quantify the workforce, while the quantity of wood or coal was estimated in kilograms. The value of the condiments was reported monthly in CFA francs on the basis of the current market price. The sheep factor was also measured in monetary terms for each *dibiterie*, according to the number of sheep bought per month and the price of the latter on the market (in CFA francs). For technical reasons related to the analytical method used, those *dibiteries* that obtain their supplies at retail and not by carcass or live animal when buying mutton, were excluded from the analysis (13/165). The depreciation value of buildings, equipment (small and heavy) and rent representing capital of *dibiteries*, energy and water and service (transport-delivery, slaughter tax) were also evaluated in CFA francs. For those buildings that belonged to the owners of the *dibiteries* while the study was conducted, a depreciation period of 25 years was fixed. For rented properties, the rent value was directly used. Small equipment (e.g. machete, grid, scale and trash) and heavy equipment (e.g. table, television and refrigerator) were amortized over 3 years, because they mostly consisted of second-hand goods. Finally, ‘other expenses’ included the monthly costs related to the purchase of cleaning and disinfection equipment and other small acquisitions such as tissues and toothpick pots.

***Output variables.*** Several types of braised products are being produced by the activity of the *dibiteries* practiced in order to gain profit, namely mutton, liver, sheep head, guts and chicken. Three outputs were selected: (i) the number of carcasses, (ii) the number of guts and (iii) the number of sheep heads sold monthly by each *dibiterie*. Thus, in the current study, we considered eight inputs allowing the production and marketing of three outputs. The descriptive statistics of the variables used are given in Table 1.

**Table 1.** Descriptive statistics of the variables used in the data envelopment analysis.

Variables	Mean	Standard Deviation	Minimum	Maximum
Inputs				
Number of personnel	2.3	1.3	1	8
Quantity of combustibles (kg)	1964	1878	90	18,000
Condiments (CFA francs)	104,442	73,869	9429	499,250
Amortization (CFA francs)	62,772	33,268	8750	284,479
Energy and water (CFA francs)	31,225	21,689	4250	236,000
Purchase of sheep (CFA francs)	1,215,076	851,979	212,500	6,375,000
Services (CFA francs)	42,937	22,085	5020	124,380
Other charges (CFA francs)	35,803	25,266	5375	145,500
Outputs				
Number of carcasses	26	26	9	225
Number of guts	725	422	225	3000
Number of sheep heads	25	26	0	225

***Regression variables.*** The identification of the determinants of the efficiency of *dibiteries* was based on the socioeconomic variables characterizing the *dibiterie* tenant and from the variables characterizing the *dibiterie*. Among these determinants, the number of years of experience of the *dibiterie* tenant is a quantitative variable. All other variables are qualitative and described in Table 2.

**Table 2.** Descriptive statistics of the socioeconomic variables of Tobit regression.

Discrete variables	Modalities	%		Assigned value
Marital status	Single	9		0
	Married	91		1
Level of education	No formal education	71		0
	Formal education	29		1
Manager status	Manager-employee	19		0
	Owner-manager	81		1
Ownership status	Not-owner	96		0
	Owner	4		1
Authorization/administrative procedure	No	56		0
	Yes	44		1
Leadership of the *dibiterie*	Individual	47		0
	Family	53		1
Visibility/brand	No signboard	61		0
	With signboard	39		1
Type of workforce	Without	35		1
	Family	49		2
	Recruited	12		3
	Mixed	4		4
Continuous variable	Mean	Std. Deviation	Minimum	Maximum
Experience (year)	18.9	9.7	0.3	46

### Analysis model

The frontier approaches such as the stochastic frontier analysis (SFA) and data envelopment analysis (DAE) to efficiency and productivity measurement have become more popular ( Tipi & Rehber, 2006). The former uses econometric methods whereas the latter uses linear programming. Furthermore, some studies found that estimation of the managerial performance in the farming sector is not neutral to methodological approach used because the scale efficiency arisen by SFA is larger than this obtained from DEA analysis. Vice versa, both methods estimate similar technical efficiency scores ( Madau, 2015). Indeed, DEA efficiency scores was expected to be less than those obtained under the specifications of SFA because the DEA approach attributes any deviation of the data from the frontier to inefficiency, while SFA acknowledges the fact that random shocks beyond the control of the farmers can affect output ( Theodoridis & Psychoudakis, 2008). However, the correlation (spearman rank coefficients) between the two approaches is positive and highly significant ( Theodoridis & Psychoudakis, 2008). There is no a priori reason therefore to expect differences in estimated efficiencies using different methods. Because, estimated differences might depend on specific data used and each model shows advantages as well as certain shortcomings relative to the other ( Madau, 2015).

In this study, the model used to estimate the technical and scale efficiency of *dibiteries* was the deterministic and non-parametric production frontier by the data envelopment analysis (DEA) under the assumption of variable returns to scale (VRS) with an input orientation. The decision on the orientation of DEA models is supported by considering the degree of a dibiterie tenant’s control over variables in the decision-making unit's production mix especially, dibiterie meat production. Indeed, *dibiterie* tenants have more control over their inputs than their outputs. The advantage of the DEA method lies in the fact that it (i) does not impose a functional form at the frontier; (ii) requires little or no information on prices and the technology used, so it requires few hypothesis; (iii) can simultaneously consider several inputs and outputs; (iv) identifies best practice and real references of inefficient firms ( Blancard *et al*., 2013). The DEA method does not allow a null value. The VRS model and the input orientation were chosen because the *dibiterie* activity in the Dakar area is exerted in an imperfect competition and the policy of sales of the *dibiteries* is more oriented towards the minimization of the factors of production (inputs) to produce a certain quality of meat. This VRS model has been proposed by Banker *et al*. (1984) and determines whether production is in an area of increasing, constant or decreasing returns. It decomposes to total efficiency (TE), pure technical efficiency (PTE) and scale efficiency (SE).

The primal equations of the VRS model in an input orientation are provided below.

$\text{Maximize}\sum_{\text{r}=\text{1}}^{\text{s}}{\text{u}}_{\text{r}}{\text{y}}_{\text{rk}}+{\text{c}}_{k}$

Under constraints

$\overset{\text{m}}{\underset{\text{i}=\text{1}}{\text{∑}}}{\text{v}}_{\text{i}}{\text{x}}_{\text{ij}}-\sum_{\text{r}=\text{1}}^{\text{s}}{\text{u}}_{\text{r}}{\text{y}}_{\text{rj}}-{\text{c}}_{k}\geq 0\;\text{j}=\text{1,}\cdots \text{,}\;\text{n}$

$\sum_{\text{i}=\text{1}}^{\text{m}}{\text{v}}_{\text{i}}{\text{x}}_{\text{ik}}=\text{1}$

${\text{u}}_{\text{r}},{\text{v}}_{\text{i}}>0\;\forall_{\text{r}}=\text{1},\cdots ,\;\text{s}\text{;}\;\text{i}=\text{1},\cdots ,\text{m}$

Where *y _rk_* is the quantity of the output r produced by the firm k; *x _ik_* is the quantity of the input *i* consumed by the firm *k*; *u _r_* is the weight of the output *r*; *v _i_* is the weight of the input *i*; *n* is the number of firms to be evaluated; *s* is the number of outputs; m is the number of inputs; *C _k_* is a measure of the returns to scale on the axes of the variables.

Because the VRS model is more flexible and envelops the data in a tighter way than the CRS model, the VRS efficiency score is equal to or greater than the CRS score ( Dhungana *et al.*, 2004). The scale efficiency (SE) score for the ith dibiterie establishments, denoted by SEi is can be calculated from the relationship of the estimate of technical efficiency (TE) of the ith dibiterie in the VRS ${(\text{TE}}_{i}^{\text{VRS}})$ and that in the CRS ${(\text{TE}}_{i}^{\text{CRS}})$ ( Theodoridis & Psychoudakis, 2008) as:

$${\text{SE}}_{i}=\frac{TE_{i}^{CRS}}{TE_{i}^{VRS}}$$

where SEi = 1.0 indicates constant returns to scale and SEi < 1.0 indicates scale inefficiency. The nature of scale inefficiency can be of two types. In order to determine the type of scale inefficiency the sum of the weights is inspected. Thus, if the sum of the weights is greater than 1.0, we have decreasing returns to scale (DRS); which means that a dibiterie is too large and belongs to the section of the frontier where decreasing returns to scale prevail. On the other hand, if the sum of the weights is less than 1.0, we have increasing returns to scale (IRS); which means that a dibiterie is too small and belongs to the section of the frontier where increasing returns to scale (IRS) prevail. Constant returns to scale occur when the sum of weights equals one ( Banker & Thrall, 1992). Finally, the percentages of dibiteries entering each of the three groups were estimated.

The technical and scale efficiency scores of the *dibiteries* were estimated using the free software DEAP 2.1/ Win4DEAP 1.1.4 developed by Coelli ( Coelli, 1996). These scores will be between 0 and 1 ( *dibiteries* that are 100% efficient reach a score of 1). The difference between the TE and the PTE scores was measured using a t-test in SPSS Statistics software version 20, at the significance level p<0.05.

The analysis of the determinants of the efficiency of *dibiteries* will allow identifying the various socioeconomic variables likely to explain the level of efficiency of the *dibiteries*, and to propose solutions to the different actors for the purpose of improvement the quality of the products. The model chosen to measure the influence of these variables is that of Tobit given the censored nature (0 to 1) of the dependent variable (efficiency scores).

Tobit model:

$\text{PT}{\text{E}}_{\text{i}}^{\text{*}}=\text{α}+{\text{β}}_{\text{i}}{\text{X}}_{i}+{\text{ε}}_{i}\;(5)$ **with** ***i*** = **1…..n ; where** PTE *_i_* **between 0 et 1**

With $\text{PT}{\text{E}}_{\text{i}}^{\text{*}}$, the dependent variable (pure efficiency), α a constant which represents the value of the intercept, *β* the vector of the coefficients affecting the explanatory variables, X _*i*_ denotes the set of explanatory variables (socioeconomic variables) and *ε _i_* is the error term of the model that differs from one observation to another.

The coefficients of the different explanatory variables were estimated using the Eviews 5.0 software (Quantitative Micro Software, LLC/4521 Campus Drive, #336, Irvine CA, 92612-2699). A variable with a positive coefficient increases technical efficiency, while a negative coefficient suggests a decreased technical efficiency of the *dibiteries* at the significance level p<0.05.

## Results and discussion

On the basis of the inclusion criteria, 165 *dibiteries* were surveyed in the Dakar region; 50 in Dakar, 50 in Pikine, 50 in Guediawaye and 15 in Rufisque. After removal of those *dibiteries* that did not comply with the conditions of the DEA method, the initial sample was reduced to 152 *dibiteries* (76%).

### Typologies of *dibiteries* and process of *dibiteries* meat production

The main characteristics of the *dibiteries* are presented in Table 3. In general, the tenants of the *dibiteries* were married men without formal education (71%) but with an average professional experience of 18.9 ± 9.7 years. Similar conditions were observed by Aw in his study on the quality of grilled meat prepared in the *dibiteries* of the Dakar region ( Aw, 1996). This study had shown that the activity of *dibiterie* is mainly exercised by men who are mostly married.

**Table 3.** Typology of *dibiteries* according to the social and economic profile of tenants.

Topics		Type of *dibiteries* (%)		
		Hausa (n = 17)	Moorish (n = 63)	Senegalese (n = 72)
Education	Yes (%)	35.3	17.5	37.5
	No (%)	64.7	82.5	62.5
Marital status	Single (%)	0.0	7.9	11.1
	Married (%)	100	92.1	88.9
Leadership of the *dibiterie*	Individual (%)	58.8	50.79	40.3
	Family (%)	41.2	49.2	59.7
Number of employees (n)	-	2.4	1.9	2.6
Experience ** (years)	-	12	21	19
Location of the *dibiterie*	Dakar (%)	64.7	27	28
	Pikine (%)	25.3	30	30
	Guediawaye (%)	15	19	42
	Rufisque (%)	-	24	-
Visibility/brand	No (%)	50	68.3	61
	Yes (%)	50	31.7	39
Ownership status	Owner (%)	0.0	2.0	11
	Non-owner (%)	100	98	89
Product	-	*dibi* Hausa	*dibi* Moorish or Senegalese	
Sale	-	Portion	Weighing (kg) and portion	
Combustible	-	Coal	Wood	

The majority of the family-type *dibiteries* (53%) and those set up with own funds (90%), were managed by their owners (81%). They reported to use mainly leased buildings (96%) and revenues from the sheep braised meat sales business meet social requirements (79%), such as health, education of children, food costs, saving for a return to the country of origin.

Three types of *dibiteries* were identified, namely Hausa, Moorish and Senegalese. The tenants of the Hausa *dibiteries* are of Nigerian nationality and Hausa ethnicity. The selling of grilled of mutton is their main activity. At the Hausa *dibiteries* the sale is usually done per portion at an average price of 1382 ± 305 CFA francs (€2.11 ± 0.46). The promoters of the Moorish *dibiteries* come from Mauritania, most of them practicing other activities, in addition to the sale of braised meat, such as trading or selling sheep (97%). The sale of the *dibi* (or sheep braised meat) was observed to be done by weight (4858 ± 329 CFA francs/kg; €7.41 ± 0.5/kg) or by portion (1000 ± 308 CFA francs; €1.52 ± 0.47), while a piece of the guts costs 100 CFA franc (€0.15). The tenants of the Senegalese *dibiteries* (72/152) are of Senegalese or Guinean origin. The sale of braised meat, which was reported to be the main activity for 91%, was based on weight (4767 ± 337 CFA francs/kg; €7.27 ± 0.51/kg) or portion at an average price of 1118 ± 380 CFA francs (€1.7 ± 0.58). All three types of *dibiteries* offer braised meat: *dibi* Hausa (440 ± 113 kg/month), *dibi* Moorish (429 ± 188 kg/month) and *dibi* Senegalese (596 ± 617 kg/month). However, the actors of the sector distinguish the *dibi* Hausa versus the *dibi* Senegalese and Moorish as two different types of products.

In the Hausa *dibiteries*, the entire carcass or cut carcass is immediately grilled with charcoal fire and regularly brushed with oil without a prior order. The well-cooked meat is served to the consumer in small pieces by adding salt, raw cut onion, mustard, pepper and *kan-kan* (cocktail of condiments consisting of peanut oilcake, chilli powder, pepper, broth, salt and garlic). In the Moorish and Senegalese *dibiteries*, the carcasses are cut and preserved entirely in the refrigerator or partially exposed in the open air in order to attract customers by its freshness. The meat is put on a wood fire only on the basis of an order. In the Moorish *dibiteries*, animal fat is added to speed up the cooking process and enhance the taste. By contrast, among Senegalese *dibiteries*, after a first round of cooking, the meat is removed from the fire and then marinated before being put back on the fire. At the end of the grilling, the meat is being cut into small pieces and served with slices of raw onion, a mixture of pepper and broth, mustard and sometimes pepper (at the request of the customer).

### Efficiency scores of *dibiteries*

Few *dibiteries* (20%) were found to be efficient according to the average global TE score of 0.74 ± 0.2 ( Table 4). In order to be 100% efficient, the management of *dibiteries* has to be optimized by reducing their inputs (resources) by 26%. The resulting margin may represent potential funding for investing in research or improving the hygienic quality of braised meat that could generate medium-term savings ( Gozlan & Marette, 2000). Indeed, reducing the consumption of these resources (inputs) saves more than a quarter (1114 CFA francs; €1.7) of the daily production cost of one kilogram of meat (4252 CFA francs/kg; €6.48/kg). This represents 16 times the estimated cost in the dairy sector (72 CFA francs; €0.11) to improve the quality of fresh milk per day in Bamako, Mali ( Bonfoh *et al*., 2006). This amount can be invested in good hygiene practices, such as hand washing, cleaning and disinfection of grilling tools, optimization of working space and training staff.

**Table 4.** Efficiency scores and returns to scale of *dibiteries*.

Efficiency score	Total efficiency	Pure technical efficiency	Scale efficiency
Means	0.74 *	0.89 *	0.83
Standard deviation	0.2	0.15	0.15
Minimum	0.316	0.523	0.33
Maximum	1	1	1
Number of *dibiteries*	152	152	152
Number of efficient *dibiteries*	31	79	31
Number of inefficient *dibiteries*	121	73	121
**RS**	**Percentage of *dibiteries* (%)**		
Increasing (IRS)	78.3		
Constant (CRS)	20.4		
Decreasing (DRS)	1.3		
Total	100.0		

*Significant difference at p < 0.05 (Student’s t-test).

On the other hand, more than half of the *dibiteries* (52%) were 100% effective as measured by PTE. The relatively high average score of 0.89 ± 0.15 may be explained by the fact that *dibiterie* tenants have easy access to inputs, particularly with regard to sheep, a key factor in production. This ease of access may be based on the relationships or contracts that the tenants have with the breeders, as well as the vicinity of the slaughterhouse. However, the PTE score being less than 1 also indicates a deficiency in the management of *dibiteries*, which may be resolved by reducing resource consumption by an average of 11% while maintaining the same level of output production.

The statistically significant difference (p<0.05) between the means of the efficiency scores TE and PTE highlights the presence of SE. Its average score of 0.83 ± 0.15 demonstrates that the *dibiteries* do not operate at their optimum size and thus save money or diseconomies of scale. However, by adjusting their size, they would reduce their inputs by 17% on average, while producing the same quantities of outputs. It is therefore necessary to invest this gain in improving the hygienic quality of braised meat.

The efficiency scores (TE and SE) obtained in this study are higher than those obtained previously in the livestock sector in Côte d’Ivoire, where TE and SE efficiencies of 0.69 and 0.87 were found for cattle production ( Youan-Bi, 2008). On the other hand, the PTE for fish producers in China was found to be 0.83 ( Sharma *et al*., 1999), 0.66 for producers of sheep in Spain ( Pérez *et al*., 2007) and 0.72 for producers of sheep in Ivory Coast ( Nuama, 2003), indicating varying levels of efficiency according to the sector of activity.

### Efficiency by type of *dibiterie*

The TE, PTE and SE were 77%, 92% and 84% for Hausa *dibiteries*, 72%, 89% and 81% for Moorish *dibiteries* and 75%, 88% and 85% for Senegalese *dibiteries*. Hausa *dibiteries* seemed to perform better than the other two types. However, no significant difference was found between the average scores of the efficiency types of these *dibiteries*. These scores indicate that it is possible to produce the same quantity of *dibi* without increasing input consumption. However, to be 100% efficient by following best practice, the Hausa, Moors and Senegalese *dibiteries* can reduce their input consumption by 23%, 28% and 25%, respectively in terms of TE; 8%, 11% and 12%, respectively in terms of PTE; and 17%, 19% and 15%, respectively in terms of SE.

### Returns to scale of *dibiteries*

The observed scale inefficiency of the *dibiteries* is at the origin of the situation of increasing returns to scale (IRS) or economies of scale of more than three-quarters of the *dibiteries* (8% Hausa, 34% Moorish, 36% Senegalese). In other words, they have not yet reached their optimal size, using too many inputs to produce relatively few outputs ( Table 4). This situation can be explained by the high purchase price of sheep and the perishable nature of the meat. Indeed, even if most *dibiteries* possess a refrigerator, the supply of sheep is organized in a way that the tenant of *dibiteries* can sell it as quickly as possible on demand of the clientele. This tense flow strategy enables them to avoid possible losses due to the irregularity of electricity and the obsolescence of the conservation equipment. In practice, to reduce input costs and to be 100% efficient, they must operate on a larger scale by increasing their size either by the number of sheep carcasses marketed or by merging with a *dibiterie* that is in a similar situation.

The analysis of the returns to scale of *dibiteries* also shows that some *dibiteries* that are efficient from a PTE point of view are also efficient overall as measured by the TE. For these, we can conclude that they therefore evolve in a situation of constant returns to scale (CRS) or they operate at their optimal size. However, few *dibiteries* (20%) are in this situation (3% Hausa, 7% Moorish, 10% Senegalese).

### Waste of inputs

To be 100% technically efficient, *dibiteries* have to increase the initial production of their output and reduce the costs associated with the input ( Table 5). In addition, the small size of the majority of these firms results in greater wastage of inputs compared with larger sized enterprises. These losses suggest that the resources used in the *dibiteries* are well above the production needs. Consequently, there might be no need to mobilize additional financial resources to address the issue of health security. Improved management of *dibiteries* would help reducing the current losses which, in turn, could contribute/to the improvement of the hygienic quality of braised meat.

**Table 5.** Percentage reduction of *dibiteries* inputs.

Variables	Average (100% efficient PTE)	Initial average	Variation
Input			Reduction (%)
Number of personnel	1.6	2.3	-30.4
Quantity of combustibles (kg)	1370	1964	-30.2
Condiments (CFA franc)	79,858	104,442	-23.5
Amortization (CFA franc)	44,768	62,772	-28.7
Energy and water (CFA franc)	21,229	31,225	-32
Purchase of sheep (CFA franc)	959,976	1,215,076	-21
Services (CFA franc)	33,294	42,937	-22.5
Other charges (CFA franc)	22,467	35,803	-37.2
**Output**			**Increase (%)**
Number of carcasses	26.1	26	+0.4
Number of guts	813	725	+12.1
Number of sheep heads	25	24.7	+1.2

PTE, pure technical efficiency.

The high consumption of energy, water and combustibles is due to the obsolescence of equipment (most notably refrigerators), the poor quality of electrical installations and the sub-optimal rationing of combustibles (wood and coal). In addition, the *dibiterie* tenants do not have any power over the price of the combustible which depends on the market. Improved kilns are known to be effective in reducing combustible consumption (20–40%) and improving product quality ( Chabi *et al*., 2014). To be efficient, *dibiteries* should reduce their energy and water consumption to 32% and the use of combustible to 30% by using improved kilns or other technologies.

*Dibiteries* rely, most of the times, on a close and easily accessible workforce. As a result, 49% of the employees at the *dibiteries* are family members and acquaintances, while only 12% of staff are recruited. Marchand (2005) believes that there is a social logic to family business operations. According to this author, the hiring within these companies is based on family preference, and it is governed mostly within the family network. This situation creating a large numeric gap between the family workforce and the recruited workforce. This situation is at the origin of a frequently encountered surplus of staff in the *dibiteries*. The remuneration of these types of workforce, however, does not differ and amounts to an average of 147,250 CFA francs/month (€224.48/month) for each *dibiterie*. In the Dakar region, 66% of the tenants of *dibiteries* employ 1 to 10 people permanently, and the remuneration is made in cash at a daily or monthly rate. Knowing that the *dibiteries* rely on average on two employees during business hours and the monthly expenditure on workforce amounts to an average of 147,250 CFA francs, the promoters of *dibiteries* pay on average a sum of 73,625 CFA francs/month (€112.24/month) for each employee. This number has significantly evolved since 1996 when the average salary of the employees of *dibiteries* was reported to be 15,000 CFA francs/month (€22.87/month) ( Aw, 1996).

### Determinants of the technical efficiency of *dibiteries*

Most socioeconomic variables do not have a relevant effect on the PTE of Dakar *dibiteries*, except for the leadership of the *dibiterie*, the ownership status, the experience of the tenants of *dibiterie* and the type of workforce ( Table 6). Table 2 shows the descriptive statistics of the variables used in this analysis.

**Table 6.** Determinants of the technical efficiency of *dibiteries*.

Variable	Coefficient (β)	Standard error	Z-statistics	Probability (p)
Manager status	-0.045	0.029	-1.515	0.129
Leadership of the *dibiterie*	-0.050 *	0.025	-1.998	0.046
Level of education	-0.033	0.025	-1.325	0.185
Marital status	-0.019	0.041	-0.486	0.627
Ownership status	0.139 *	0.058	2.419	0.016
Experience	0.002 *	0.001	2.047	0.041
Authorization/administrative procedure	-0.013	0.022	-0.599	0.549
Visibility/brand	0.025	0.023	1.075	0.282
Type of workforce	-0.039 *	0.009	-4.021	0.000
Log Likelihood	92.409			

*Significant difference at p < 0.05.

The analysis of the determinants demonstrated that the managerial performance of the *dibiteries* is negatively influenced by a heavy family involvement which seems to render the establishments technically less efficient. The fact that the administration of these *dibiteries* is entrusted to multiple people is a factor favoring poor management of outputs and profits. In family enterprises, the existence of social relations of mutual aid and solidarity often lead to an environment that fosters credits to customers, donations and self-consumption. Low investment is often combined with a large part of the profits going towards family care ( Marchand, 2005) instead of being used to improve the quality of the products sold. Similarly, the loyalty of a customer is linked to the network of family or community where, sometimes, sales are at a loss for social reasons. It is the very same networks which, in the event of a working capital deficit or bankruptcy, provide the *dibiterie* tenant with the necessary funds for the resumption of activity ( Marchand, 2005). The learning and the transmission of the knowledge of the trade happens from generation to generation, with an important recourse to the family ( Marchand, 2005). The employee coming from a family network will not have an obligation of delivering results compared to a non-related employee recruited. Workforces with a family apprenticeship are prone to generate a typical product of organoleptic quality that is highly appreciated by the consumers but of poor quality with regard to hygiene. Some basic hygiene is needed and the workers caught in the family do often not have adequate training. However, collective catering is a profession and a métier, and requires appropriate training regardless of the origin of the workforce. The informal sector must therefore adapt to conventional methods when training the family worker force.

The ownership status was found to have a positive effect on the technical performance of the *dibiteries*, demonstrating that renting the place of establishment leads to better results and that the revenue figure allows covering production costs and rent. However, the *dibiterie* tenant that is renting tends to invest little in the improvement of hygiene in the *dibiterie*, in order to avoid the risk of breaching the lease before amortization of investments in the premises. This is a major constraint to be taken into account for achieving the twofold objective of improving both the microbiological quality of *dibiterie* meat and the technical performance of these establishments.

The amount of experience of the tenant, on the other hand, has a positive effect on the technical performance of the *dibiteries*. The seniority in exercising this duty is favorable to the performance of the company, because experienced *dibiterie* tenants acquire a certain ease of negotiating the prices of the factors of production compared with the less experienced ones.

The manager status, the level of education, the marital status of the tenant, the presence of an indicative sign (visibility/brand) and the administrative procedures did not seem to have a significant influence on the managerial performance of the *dibiteries*.

## Conclusion

With average efficiency scores less than 1, *dibiteries* are inefficient technically and in terms of scale. In general, the global technical inefficiency of around 26% observed, seemed more related to scale inefficiency than to pure technical inefficiency. This situation is due to the fact that the majority of these *dibiteries* are in situation of increasing returns to scale by using more production inputs (resources for braised meat production) for a low level of production (braised meat sold). Thus, these dibiteries that are operating scale inefficiently are doing so because of the not adapted size of their operations rather than because of they use technically inefficient production mixes. Only 20% evolve and operate in a situation of constant returns to scale. However, by following best practices, *dibiteries* can make gains by reducing the consumption of their input factors, while producing the same quantity of outputs. These gains could be used for training good hygiene practices on handwashing, cleaning and disinfecting grilling tools, optimizing work space and training staff.

The analysis of the determinants of the TE of *dibiteries* shows that the ownership status and the tenants' experience improve the managerial performance of these companies. On the other hand, the family-run nature of the *dibiterie* and the type of workforce significantly reduce the PTE of the *dibiteries*. It is therefore recommended to re-organize the *dibiterie* activities according to defined technical, material and financial support frameworks. Support is needed by the *dibiteries* owners in training, financial and economic analysis, facilitation of access to dedicated spaces, professional organization and credit.

## Data availability

### Underlying data

Open Science Framework: Spreadsheet of the answers to questions in the questionnaire. https://doi.org/10.17605/OSF.IO/CVSZU ( Orou-Seko *et al*., 2019)

This project contains answers to each question from each of the *dibiteries*.

### Extended data

Open Science Framework: Underlying data of typology, TE and SE of dibiteries in Dakar, Senegal. https://doi.org/10.17605/OSF.IO/Y6NSF ( Orou-Seko *et al*., 2019a).

This project contains the following underlying data:

- Efficiency_Scores.csv (results of efficiency analysis)
- Projected_value.csv (data on factors relating to costs)
- Variables_Tobit regression.csv (variables used in Tobit model)

Open Science Framework: Questionnaire administered to the dibiteries owners in Dakar, Senegal. https://doi.org/10.17605/OSF.IO/G6F2U ( Orou-Seko *et al*., 2019b).

This project contains an English translation of the questionnaire administered to the owners of *dibiteries*.

Data are available under the terms of the Creative Commons Zero "No rights reserved" data waiver (CC0 1.0 Public domain dedication).

## References

[ref-1] Aw A: Contribution à l’étude de la qualité des viandes grillées préparées dans les *dibiteries* (grilladeries sénégalaises) dans la région de Dakar. Dakar, Thèse Méd. Vét., EISMV, Dakar.1996;106 Accessed 10th November 2016. Reference Source

[ref-2] Banker RD Charnes A Cooper WW: Some models for estimating technical and scale inefficiencies in data envelopment analysis. *Management Sci.* 1984;30(9):1078–1092. 10.1287/mnsc.30.9.1078

[ref-26] Banker RD Thrall RM: Estimation of returns to scale using data envelopment analysis. *Eur J Oper Res.* 1992;62(1):74–84. 10.1016/0377-2217(92)90178-C

[ref-3] Blancard S Boussemart JP Flahaut J: Les fonctions distances pour évaluer la performance productive d’exploitations agricoles. Paris, SFER, *Eco Rur.* 2013;334:7–22. 10.4000/economierurale.3887

[ref-4] Bonfoh B Roth C Traoré AN: Effect of washing and disinfecting containers on the microbiological quality of fresh milk sold in Bamako (Mali). *Food Control.* 2006;17(2):153–161. 10.1016/j.foodcont.2004.09.015

[ref-5] Chabi NW Konfo CTR Emonde PDM: Performance d’un dispositif amélioré de fumage (four Chorkor) sur la qualité du poisson fumé dans la commune d’Aplahoué (Sud-est du Bénin). *Int. J of Innovation and Applied Studies.* 2014;9(3):1383–1391. Reference Source

[ref-6] Coelli TJ: A Guide to DEAP Version 2.1: A Data Envelopment Analysis (Computer) Program. *Armidale.*CEPA Working Paper 96/08.1996;49 Reference Source

[ref-60] Dhungana BR Nuthall PL Nartea GV: Measuring the economic inefficiency of Nepalese rice farms using data envelopment analysis. *Aust J Agric Resour Econ.* 2004;48(2):347–369. 10.1111/j.1467-8489.2004.00243.x

[ref-7] Dione A: Contribution à l’étude de la qualité bactériologique de quelques denrées alimentaires d’origine animale commercialisée sur le marché Dakarois. Dakar, Thèse Méd. Vét., EISMV, Dakar.2000;120 Accessed 28th November 2017. Reference Source

[ref-8] Duho KSD: Le nettoyage et la désinfection en restauration collective à l’hôpital principal de Dakar. Dakar, Thèse Méd. EISMV, Dakar.2012;143 Accessed 28th November 2017. Reference Source

[ref-9] Duteurtre G: Evolution du secteur de l’élevage ouest africain. *Grain de sel.* 2009;46–47:12–15. Reference Source

[ref-10] Gozlan E Marette S: Commerce international et incertitude sur la qualité des produits. *Rev Eco Int.*(CEPII).2000;81:43–63. Reference Source

[ref-27] Madau FA: Technical and Scale Efficiency in the Italian Citrus Farming: Comparison between SFA and DEA Approaches. *Agricultural Economics Review.* 2015;16(2):15–27. 10.22004/ag.econ.253696

[ref-11] Mankor A: Consommation urbaine de viandes en Afrique de l’Ouest: l’exemple de Dakar. *In*: le dossier: Evolution de l’élevage ouest-africain. *Grain de sel.* 2009;46–47:16–17. Reference Source

[ref-12] Marchand G: Economie informelle au Sénégal. Logique de fonctionnement de quelques entreprises informelles à Saint-Louis. Mémoire de master en sociologie, Université Laval, Quebec.2005;130 Accessed 5th December 2017. Reference Source

[ref-13] Ndoye F Diop A Sokhona K: Evolution des styles alimentaires à Dakar. Centre de coopération internationale en recherche agronomique pour le développement (CIRAD), Enda-Graf.2001;63 Accessed 16th January 2017. Reference Source

[ref-14] Nuama E: Évaluation de la performance productive des exploitations ovines en Côte-d’Ivoire. *Revue Ivoirienne des Sciences Economiques et de Gestion (RISEG).* 2003;9(1):65–79.

[ref-15] Orou-Seko M Ossebi W Traoré GS: Spreadsheet of the answers to questions in the questionnaire.2019 10.17605/OSF.IO/CVSZU

[ref-16] Orou-Seko M Ossebi W Traoré GS: Underlying data of typology, TE and SE of *dibiteries* in Dakar, Senegal. *OSF.* 2019a 10.17605/OSF.IO/Y6NSF PMC7185242 32382700

[ref-17] Orou-Seko M Ossebi W Traoré GS: Questionnaire administered to the dibiteries owners in Dakar, Senegal. *OSF.* 2019b 10.17605/OSF.IO/G6F2U

[ref-18] Pérez JP Gil JM Sierra I: Technical efficiency of meat sheep production systems in Spain. *Small Ruminant Res.* 2007;69(1–3):237–241. 10.1016/j.smallrumres.2006.02.003

[ref-19] Sharma KR Leung P Chen H: Economic efficiency and optimum stocking densities in fish polyculture: an application of data envelopment analysis (DEA) to Chinese fish farms. *Aquaculture.* 1999;180(3–4):207–221. 10.1016/S0044-8486(99)00202-1

[ref-20] Sénégal, Ministère de l’économie, des finances et du plan: Situation économique et sociale du Sénégal en 2015. Dakar: ANSD.2018;13, Accessed 10th January 2018. Reference Source

[ref-21] Sénégal, Ministère de l’économie, des finances et du plan: Recensement Général de la Population et de l’Habitat, de l’Agriculture et de l’Elevage (RGPHAE). Rapport définitif. Dakar: ANSD.2014;417, Accessed 16th June 2017. Reference Source

[ref-22] Sénégal, Ministère de l’Elevage et des Productions Animales: Rapport de revue du secteur de l’élevage. Dakar.2017;33, Accessed 16th January 2018. Reference Source

[ref-28] Theodoridis AM Psychoudakis A: Efficiency Measurement in Greek Dairy Farms: Stochastic Frontier Vs. Data Envelopment Analysis. *International Journal of Economic Sciences and Applied Research.* 2008;1(2):53–67. Reference Source

[ref-23] Thornton P Herrero M Freeman A: Vulnerability, climate change and livestock-research opportunities and challenges for poverty alleviation. *Journal of SAT of Agricultural Research.* 2007;4(5):1–23. Reference Source

[ref-29] Tipi T Rehber E: Measuring technical efficiency and total factor productivity in agriculture: the case of the South Marmara region of Turkey. *New Zeal J Agr Res.* 2006;49(2):137–145. 10.1080/00288233.2006.9513703

[ref-24] Youan-Bi A: Efficacité managériale des éleveurs de bovins de Côte d’Ivoire: cas des départements de Toumodi et de Korhogo. Abidjan, Thèse Doct. (PhD), Université de Cocody.2008;166.

[ref-25] Yougbare B: Appréciation des risques de contamination microbienne de la viande de petits ruminants dans les abattoirs et les *dibiteries* de Dakar, Sénégal. Dakar, Mémoire de Master en santé publique vétérinaire, Ecole Inter-Etats des Sciences et / Médecine Vétérinaires de Dakar (EISMV).2014;32, Accessed 08th January 2016. Reference Source

