# Supplementary material for: Typology, technical efficiency and scale economy of dibiteries in Dakar, Senegal
Source: AAS Open Res. 2019 Dec 12;2:10. Originally published 2019 Mar 11. [Version 2] doi: 10.12688/aasopenres.12953.2 (PMC7185242; doi:10.12688/aasopenres.12953.2)
Supplement: Peer review history

# Peer review: Reviewer response for version 2

**DOI:** 10.21956/aasopenres.14134.r27316

**Published:** 2020-01-07

**Authors:** Fabio A. Madau

**Affiliations:** 1 Department of Agriculture, University of Sassari, Sassari, Italy

Considering the improvements made following the suggestions formulated by the referee, I think that the paper is suitable for publication in this form.

I confirm that I have read this submission and believe that I have an appropriate level of expertise to confirm that it is of an acceptable scientific standard.

# Peer review: Reviewer response for version 1

**DOI:** 10.21956/aasopenres.14031.r27283

**Published:** 2019-12-05

**Authors:** Tolga Tipi

**Affiliations:** 1 Faculty of Agriculture, Department of Agricultural Economics, Bursa Uludag University, Bursa, Turkey

- This manuscript focused on estimating technical efficiency and scale economy using the DEA method of the dibiteries in Dakar. Also, a Tobit model was used to identify the managerial determinants of dibiterie efficiency.
- There are many studies undertaken in the agriculture and food industry that measure efficiency and factors affecting efficiency using the DEA and Tobit models. As far as I am concerned, the efficiency analysis of dibiteries was conducted for the first time. Therefore, this research is original and contributes to the literature.
- One of the main aims of this manuscript was to assess the managerial performance of the dibiteries. This aim seems to have been addressed. However, further explanation that supports how to achieve scale economy of the dibiteries is needed in the conclusions section.
- The manuscript claims in "the sampling and data collection" section that 200 dibiteries were chosen from 4 provinces of Dakar representing 50 dibiteries from each province. However, the "results and discussion section" states that 165 dibiteries were surveyed; 50 from three provinces and 15 from the fourth province. In contrast, the "abstract" states that 163 dibiteries were surveyed. There seems to be a conflict between the statements and this needs clarifying. 

I confirm that I have read this submission and believe that I have an appropriate level of expertise to confirm that it is of an acceptable scientific standard.

# Response

**Published:** 2019-12-06

**Authors:** Malik Orou Seko

**Affiliations:** Ecole Inter-Etats des Sciences et Médecine Vétérinaires (EISMV), Senegal

**I would like to thank you for your valuable inputs on our paper. All comments have been carefully assessed and were taken into consideration during the revision to improve the quality of the manuscript.**

**Please find below the response to your comments indicating the corresponding changes in the manuscript.**

One of the main aims of this manuscript was to assess the managerial performance of the dibiteries. This aim seems to have been addressed. However, further explanation that supports how to achieve scale economy of the dibiteries is needed in the conclusions section.

**Response:**

**A paragraph has been added at the conclusion level to explain how the dibiteries achieve scale economy.**

The manuscript claims in "the sampling and data collection" section that 200 dibiteries were chosen from 4 provinces of Dakar representing 50 dibiteries from each province. However, the "results and discussion section" states that 165 dibiteries were surveyed; 50 from three provinces and 15 from the fourth province. In contrast, the "abstract" states that 163 dibiteries were surveyed. There seems to be a conflict between the statements and this needs clarifying.

**Response:**

**Initially, there were accidental choices of 200 dibiteries. But at the end of the investigations we obtained 165, i.e. 82% of the planned sample. However, given the specifications of the DEA model, this size was reduced to 152 dibiteries, i.e. 76% of the planned sample. The corresponding figure has been corrected in the summary, i.e. 152 dibiteries instead of 165.**

**I hope that the revised manuscript is now suitable for publication in *AAS Open Research*, and look forward to hearing from you. Thank you.**

# Peer review: Reviewer response for version 1

**DOI:** 10.21956/aasopenres.14031.r27038

**Published:** 2019-07-30

**Authors:** Fabio A. Madau

**Affiliations:** 1 Department of Agriculture, University of Sassari, Sassari, Italy

The paper is focused on estimating technical and scale efficiency in "dibiteries" of the region around Dakar (Senegal). Although the methodology has been widely applied in the economic literature - especially in agriculture and agro-food sectors - the paper appears sufficiently original because it is one, or the unique, case of efficiency analysis applied at this sort of activity. Objectives are clearly illustrated, methodology is correctly applied, results are well showed, and discussion is congruent with findings.

 In my opinion, the paper is suitable for publication after the authors provide a clarification about the choice of the method. Specifically, DEA is correctly applied, but the rationale underlined that suggests a non-parametric approach rather than a parametric one is not clear. Choice should be illustrated more in-depth - especially concerning the estimation of inefficiency effects - also citing sono comparation analyses is useful for better justification of it (see Theodoridis and Psychoudakis, 2008 ^1^ and Madau, 2015 ^2^).

 I suggest publication after the authors illustrate why DEA was considered preferable to other methods (e.g., Stochastic Frontier Analysis).

I confirm that I have read this submission and believe that I have an appropriate level of expertise to confirm that it is of an acceptable scientific standard, however I have significant reservations, as outlined above.

## References

[1] : Efficiency Measurement in Greek Dairy Farms: Stochastic Frontier Vs. Data Envelopment Analysis. *International Journal of Economic Sciences and Applied Research*.2008;1(2) :53-67 Reference source

[2] : Technical and Scale Efficiency in the Italian Citrus Farming: A Comparison between SFA and DEA Approaches. *Agricultural Economics Review*.2015;16(2) :15-27 Reference source

# Response

**Published:** 2019-12-06

**Authors:** Malik Orou Seko

**Affiliations:** Ecole Inter-Etats des Sciences et Médecine Vétérinaires (EISMV), Senegal

In my opinion, the paper is suitable for publication after the authors provide a clarification about the choice of the method. Specifically, DEA is correctly applied, but the rationale underlined that suggests a non-parametric approach rather than a parametric one is not clear. Choice should be illustrated more in-depth - especially concerning the estimation of inefficiency effects - also citing sono comparation analyses is useful for better justification of it (see Theodoridis and Psychoudakis, 2008 ^1^ and Madau, 2015 ^2^)

**Response:**

**I would like to thank you for your valuable inputs on our paper. All comments have been carefully assessed and were taken into consideration during the revision to improve the quality of the manuscript. Please find below the response to your comments indicating the corresponding changes in the manuscript.**

**At the analysis model section, we have listed and presented the links of the efficiency scores obtained when using the two approaches to the estimation of managerial performance of firms (DEA and Stochastic Frontier Analysis – SFA). Then, we justified more in-depth why we preferred to use DEA rather than SFA. Two other paragraphs have also been added in the same section, concerning the estimation of inefficiency effects (TE and SE).**

**I hope that the revised manuscript is now suitable for publication in *AAS Open Research*, and look forward to hearing from you. Thank you.**
